# A new concept for implant-borne dental rehabilitation; how to overcome the biological weak-spot of conventional dental implants?

**DOI:** 10.1186/s13005-017-0151-3

**Published:** 2017-09-29

**Authors:** Nils-Claudius Gellrich, Björn Rahlf, Rüdiger Zimmerer, Philipp-Cornelius Pott, Majeed Rana

**Affiliations:** 1 Department of Oral and Maxillofacial Surgery, Hannover Medical School, Carl-Neuberg-Str. 1, 30625 Hannover, Germany; 2 Department of Prosthetic Dentistry and Biomedical Materials Research, Hannover Medical School, Carl-Neuberg-Str. 1, 30625 Hannover, Germany

**Keywords:** CAD/CAM, Dental implants, Subperiosteal implants, Digital workflow, Bone defects

## Abstract

**Background:**

Every endosseous dental implant is dependent on an adequate amount and quality of peri-implant hard and soft tissues and their fully functional interaction. The dental implant could fail in cases of insufficient bone and soft tissues or due to a violation of the soft to hard tissues to implant shoulder interface with arising of a secondary bone loss.

**Method:**

To overcome this biological weak-spot, we designed a new implant that allows for multi vector endosseous anchorage around the individual underlying bone, which has to be scanned by computed tomography (CT) or Cone beam CT (CBCT) technique to allow for planning the implant. We developed a workflow to digitally engineer this customized implant made up of two planning steps. First, the implant posts are designed by prosthodontic-driven backward planning, and a wireframe-style framework is designed on the individual bony surface of the recipient site. Next, the two pieces are digitally fused and manufactured as a single piece implant using the SLM technique (selective laser melting) and titanium-alloy-powder.

**Results:**

Preoperative FEM-stress-test of the individual implant is possible before it is inserted sterile in an out-patient procedure.

**Conclusion:**

Unlike any other historical or current dental implant protocol, our newly developed “individual patient solutions dental” follows the principle of a fully functional and rigid osteosynthesis technology and offers a quick solution for an implant-borne dental rehabilitation in difficult conditions of soft and hard tissues.

## Background

A successful dental implant is based on optimal hard and soft tissue requirements. It includes appropriate dimensions (vertical, sagittal, and transverse) and quality of the bone together with healthy soft tissues around the implant shoulder including an area of non-mobile keratinized gingiva.

Appropriate dental implant planning and treatment has to envision long-term success. This entails prevention of adverse effects such as peri-implantitis with secondary bone loss around the implants and consecutive long-term failure of the implants [2, 19]. One of the modern developments in implant dentistry has been the downsizing of the length and diameter of conventional dental implants, thereby, improving their surfaces and biomaterials used, to allow for the “shorter and thinner approach” [15]. The only widely accepted real different design in implant dentistry is the long zygomaticus implant [18], which is limited to the maxilla, and anchors far away from the oral cavity in the ipsilateral malar bone.

With our new concept for implant-borne dental rehabilitation, we focus on the idea of a digital workflow for implant planning (prosthodontic backward), engineering, and manufacturing and have revisited the subperiosteal implants designed and installed in the 1940s [10]. In the late 1980s and early 1990s, CAD/CAM techniques were used to avoid the extra surgical step to take direct bone-impressions of patients [4, 8, 11, 22] and to improve the peri-implant bone-healing [1, 13]. However, the combination of a perfectly fitting subperiosteal implant together with a rigid fixation technique has not been adequately considered.

Considering the advantages and disadvantages of the conventional cylindrical or conical dental implants and the subperiosteal implants, we have developed a new design for difficult cases, where the quantity and quality of the bone, as well as the surrounding soft tissues, do not match the requirements for conventional implant dentistry. To overcome the biological weak-spot of regular dental implants, we designed a new implant that allows for multi vector endosseous anchorage around the individual underlying bone. We developed a workflow to digitally engineer this customized implant made up of two planning steps. First, the implant posts are designed by prosthodontic-driven backward planning**,** and a wireframe-style framework is designed on the individual bony surface of the recipient site. Next, the two pieces are digitally fused and manufactured as a single-piece implant by the SLM-technique out of a titanium-aluminum-vanadium alloy (Ti6Al4V specified according to ASTM F136-02a (ELI Grade 23)). Preoperative FEM-analysis of the individual implant is possible before it is inserted in a sterile manner in an out-patient procedure.

**Fig. 1:**
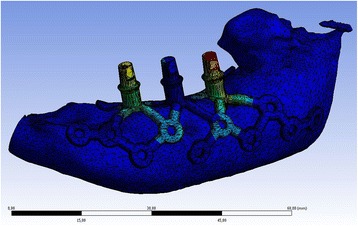
Computer-simulation of occlusal loading of the IPS-d (Any Body Repository 1.6 Ansys-Workbench) – stress test with a color map: red is high, yellow is medium, and green is low stress, analyzed with regard to a worst case scenario of a maximum clinical loading stress of 440 N

**Fig. 2:**
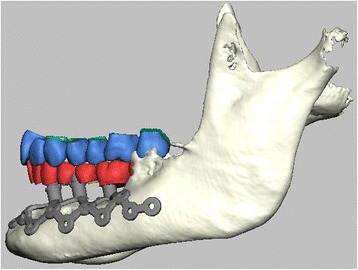
Lateral view of the IPS-dental on the left atrophic mandible (the same framework as shown in Fig. 1) during the planning process; the anchorage is separated from the soft tissue around the implant posts, to improve the (multi-vector) fixation and to allow for a one-fit-only and exact positioning during the surgery, the posts are aligned according to the virtually inserted dental arches

In the cases where Individual Patient Solution-dental (IPS-d) is considered, the soft tissues play a significant role due to quantitative and qualitative compromise including the post-irradiation damage [16]. If available, in cases of oral malignancies, pre-ablative CT or cone beam scan is used for digital planning. Impressions and cast models prior to resection are used as a reference, laser scanned, and virtually inserted into the 3D–dataset. Alternatively, an impression is taken over a wax-up, and cast models are produced, laser scanned, and used for prosthodontic backward planning. Both jaws have to be considered when planning the adequate vectors for the complex one-piece IPS-d in order to accurately transfer the individual interocclusal relationships. CoDiagnostiX® Version 9.7.1 (Dental Wings GmbH, Chemnitz, Germany) software is used for positioning the cylindric posts similar to the planning of conventional dental implants. STL-Export of the implant position is exported into the software Geomagic FreeForm Plus (333 Three D Systems Circle, Rock Hill, SC 29730, USA). Here, similar to the already published method of customized orbital implant planning, a digital framework as a footplate is created (its design is inspired by the railroad crossing sign) and projected on top of the CT- or cone beam CT-scan of the anatomy at the defect site. The arms for retention of the implant are extended anteriorly and posteriorly as well as lingually (vs. palatally) or buccally to allow for adequate screw-hole retention. The geometry, position, and shape of the implants can be completely individualized, with as many screw-holes as possible to achieve rigid fixation, especially in the well-known buttresses of the maxilla (lateral and medial midfacial pillars) or the mandible. Together with the individualized footplate-framework, the vertically designed posts are digitally unified and connected to a single-piece implant. Before the digitally engineered implant is inserted, an FEM-stress-test (Ansys Workbench 16.2®, ANSYS, Inc., Southpointe 2600 ANSYS Drive, Canonsburg, PA 15317, USA) is performed (Fig. 1). Due to the multistep-manufacturing-processes (selective laser melting, heating, glass particle blasting, milling, finishing) and the related influences on the materials, the conventional FEM stress testing was not used. Instead a worst case scenario using a maximum clinical loading stress of 440 N was used. The digital prosthodontic-backwards planned protocol is illustrated in Fig. 2 for the left mandibular body. Following digital planning and data transfer, the IPS-dental was finally manufactured using the selective laser melting technique (KLS Martin Group, Tuttlingen, Germany).

## Method

**Fig. 3:**
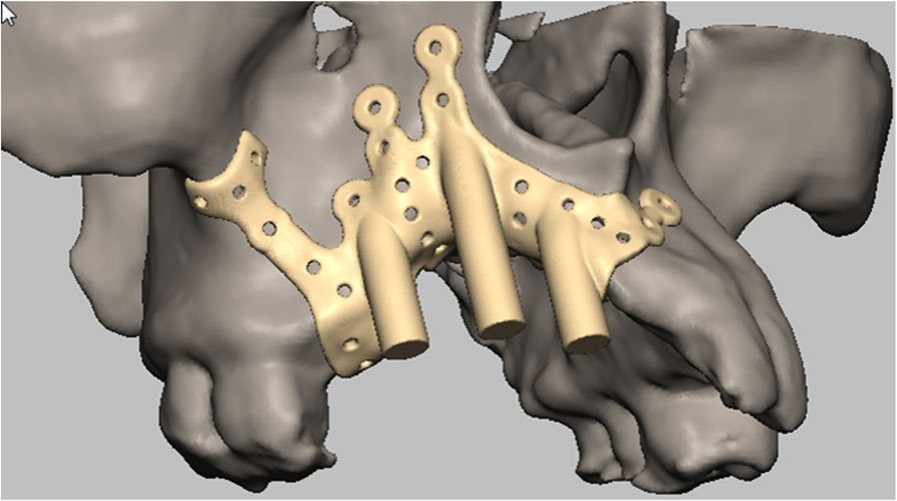
Post-traumatic right maxillary reconstruction with an IPS-dental, that is designed to anchor in the midfacial buttresses and subnasal area; multiple screw holes allow for multi vector rigid fixation. The posts can be modified to any geometry

We describe the technical specification using another case for the right maxilla (Fig. 3):

The framework is designed as follows: First, the implant posts are defined and positioned according to the prosthodontic backward planning. The framework is designed depending on the underlying bone thickness and the vector of insertion of the later supra structure. Finally, the transition zone between the posts and the framework is reinforced and the final implant design is checked for undercuts, which should be avoided to prevent gaps vs. no-contact between framework and bone. Since the posts include the functionality of the regular dental implant abutments, no additional technical interface is needed for further prosthodontic treatment. Therefore, our implant posts primarily are exposed to the oral cavity as a non-submerged technique.

The framework thickness is 1.2 mm, the screw-holes are 2.0 mm in diameter, and a countersink design allows the screw head to sit flush with the outer surface of the metallic framework. Any complex anatomy of the recipient site can be addressed design-wise and reflected in the individual patient solution design, for example, openings in the framework can be designed to prevent interference with the mental nerve. Multiple screw holes are designed in the skeletonized metallic framework to allow for a functionally stable load bearing implant with a high stability provided by multi vector mini-screw retention. The number of screws required depends on the size of the implant, with a minimum of 15 screws and a maximum of 30 screws.

The post diameter is 4 mm; the vector is adjusted according to the opposite jaw dentition taking into account the planned type of supra structure. We favor a removable partial prosthesis locked onto a metal bar or on telescopes. The prosthodontic height is designed backward starting from the occlusal level and is strongly dependent on the individual soft tissue level. In cases of extremely low interocclusal space, the suprastructure design could be a ball-connection only. A rotation-stable telescoping design is favored as it allows for the well-established manufacturing of individual dental restorations in the usual workflow with a standard dental lab. The precision in digital planning and engineering add to a straight forward insertion with a clear one-fit-only approach, so that mistakes due to malpositioning of the implant in terms of the vector, vertical, transverse, and sagittal positions are avoided.

## Results

We report the IPS-dental planning and engineering from scratch for each patient, with the intention to optimize the planning process by using digitally adjustable and deformable STL-models for the dental arch instead of laser-scanning the wax-up. Furthermore, STL-models to be digitally projected – comparable to our approach in engineering customized orbital implants for one- to four-wall-orbital defect reconstructions – on top of the individual maxillary and mandibular anatomy are designed. To date, the planning process typically requires 4–6 h for a medical engineer; SLM-manufacturing takes 6 days including shipping of the implant; it is delivered together with a polyamide-printed-out-3D-model of the recipient site so that a physical model of the recipient site is available before and during surgery. Finishing procedure prior to autoclaving the individual patient solution dental is to polish the posts starting from the framework. The implant is autoclaved in the sterilization department. Exposure of the recipient site includes a lateral incision to the latter implant-post perforation through the overlying mucosa or soft tissues. This is intended to save the soft tissues around the alloplastic implant. Intraoperatively, a single shot of penicillin-G is administered, and postoperatively, a panoramic radiograph is taken. The soft tissues are allowed to settle for 6 weeks prior to prosthodontic suprastructure manufacturing.

## Discussion

Our presented technique is based on the long-time proven knowledge of rigid fixation used in cases of craniomaxillofacial trauma, tumor, and reconstructive surgery [7, 17]. However, the concept has not been applied in the field of subperiosteal implant treatment. Our new single step IPS-dental concept utilizes well-known materials with known biocompatibility [9, 12], the advantage of digital engineering as a prosthodontic backwards plan and manufacturing, resulting in an innovative protocol and an out-patient procedure for difficult dental implant cases [14]. The proposed new method is not to replace the standard dental implants but to provide a single step alternative procedure for patients who cannot undergo the regular augmentation-implant insertion-free mucosal grafting protocols due to severe compromise in bone and soft tissue quantity and quality. The new procedure also helps avoid long-lasting, more invasive, and costly procedures with multiple surgical steps, where, in the end, the weak-spot of the regular dental implant remains or is even more worse [5].

Non-rigidly fixed subperiosteal implants that have been used previously lead to progressive bone loss due to movement of the framework and the underlying bone, resulting in bone-atrophy and worsening of the anatomic situation. Our new IPS-dental utilizes the reliable technique of rigid fixation, which is known since the 1950s and is a standard in traumatology and reconstructive surgery today [6].

The craniomaxillofacial skeleton atrophy below the underlying bone of rigidly fixed plates is uncommon, though long term (over 5–10 years) studies of IPS-dental must be performed to confirm this. Atrophy per se would not be a reason for the failure of this implant as long as the rigid fixation of the implant-framework itself is given. Unlike the conventional reconstruction protocols used for larger jaw-defects, wherein distant donor sites such as the iliac crest, fibula, and scapula are harvested for bone, and where the bone has to be placed in an appropriate position to allow for a later favorable dental implant positioning and prosthodontic rehabilitation, our technique does not lead to additional comorbidities due to a second operation site. Conventional dental implant-borne rehabilitation protocols strongly depend on the appropriate soft-tissue to hard-tissue to the implant-shoulder interface. Peri-implantitis and subsequent bone loss can directly reduce the bony anchorage and lead to failure of the implant. However, the implant-shoulder interface is directly at the point of entry of the implant axis into the bone, i.e., the implant axis resembles the axis of bony anchorage [20]. The IPS-dental avoids this problem of internal fixation at the area of mucosal penetration by taking the fixation points and axes of the screw fixation away and far away from the soft tissue around the implant posts. Therefore, inflammation around the implant posts does not lead to an immediate influence on bony fixation of the IPS-dental. The advantage of the bony anchorage far away from the oral cavity is already known and used with the zygoma implants [3]. These implants anchor in the malar bone, on their way from the implant shoulder in the oral cavity to the anchoring apical part, and this device is in close vicinity or even penetrates through the maxillary sinus. One drawback is the limited indication, i.e., they address the lateral maxilla only. However, similar to the IPS-dentals, they also have the advantage of the bony anchorage far away from the implant shoulder [18].

The compromised soft tissues and hard tissues always pose a challenge to the conventional implant borne dental rehabilitation protocols, which are further aggravated in situations of radiotherapy, immunosuppression, tissue loss, scarring, deformity, etc. In these cases, peri-implantitis leads to progressive loss of the conventional dental implants [21]. Therefore, there is a definite need to offer a long term treatment to these patients that basically can be handled as an outpatient procedure with immediate loading.

## Conclusion

Dental rehabilitation in patients with severe atrophy using IPS-dental might be a potential strategy to overcome the well-known problems of conventional implantology.
